# Enhancing CD8^+^ T Cells Infiltration Through the Protein Arginine Methyltransferase 5 (PRMT5)/CXCL10 Axis Restricts Cervical Cancer Progression

**DOI:** 10.3390/biom15121717

**Published:** 2025-12-10

**Authors:** Yongshuai Jiang, Yingying Wei, Ziyang Li, Zhenghang Huang, Junsheng Dong, Weijuan Gong, Li Qian

**Affiliations:** 1 School of Basic Medical Sciences & School of Public Health, Faculty of Medicine, Yangzhou University, Yangzhou 225009, Chinawjgong@yzu.edu.cn (W.G.); 2 Key Laboratory of the Jiangsu Higher Education Institutions for Nucleic Acid & Cell Fate Regulation, Yangzhou University, Yangzhou 225009, China; 3 Jiangsu Key Laboratory of Zoonosis, Yangzhou University, Yangzhou 225009, China; junsheng@yzu.edu.cn; 4 College of Veterinary Medicine, Yangzhou University, Yangzhou 225009, China

**Keywords:** PRMT5, cervical cancer, CXCL10, CD8^+^ T cells, tumor microenvironment

## Abstract

PRMT5, a type II methyltransferase catalyzing symmetric dimethylation of arginine residues, has emerged as a promising therapeutic target in various cancers. However, the precise mechanism by which PRMT5 mediated the tumor immune microenvironment, particularly CD8^+^ T cell recruitment in cervical cancer remains elusive. Analysis of data from The Cancer Genome Atlas (TCGA) revealed elevated PRMT5 mRNA levels in cervical cancer tissues, which correlated with reduced immune cell infiltration and poorer patient prognosis. To further investigate the role of PRMT5 in tumor development, a CD8 knockout (KO) mouse tumor model was utilized. Significant inhibition of tumor growth was observed in cervical cancer using a mouse model lacking PRMT5. Notably, this antitumor effect was attenuated in CD8 KO mice lacking functional CD8^+^ T cells. Mechanistically, RNA sequencing (RNA-seq) analysis was conducted to explore how PRMT5 regulates immune cell recruitment. Disruption of PRMT5 was found to increase the secretion of chemokine CXCL10 by tumor cells. CXCL10 binds to its receptor CXCR3, thereby recruiting T cells to the tumor. Furthermore, in CXCR3 KO mice, PRMT5 knockdown failed to enhance T cell infiltration into tumors. These findings indicate that PRMT5 knockdown promotes CD8^+^ T cell recruitment to the tumor microenvironment via CXCL10 signaling. Furthermore, the therapeutic efficacy of the selective PRMT5 inhibitor EPZ015666 was evaluated in a cervical cancer xenograft mouse model. Treatment with EPZ015666 effectively suppressed tumor growth. In summary, these findings elucidate a novel mechanism whereby PRMT5 depletion in cervical cancer cells triggers a CXCL10-mediated chemotactic response, enhancing CD8^+^ T cell infiltration and restricting tumor progression. Thus, our study provides compelling evidence supporting the potential targeting of PRMT5 as a viable immunotherapeutic strategy for cervical cancer.

## 1. Introduction

Cervical cancer represents one of the most prevalent gynecological malignancies globally, accounting for approximately 6.5% of all cancers in women [1]. Early detection and appropriate staging are pivotal for improving life expectancy [2,3]. In addition to surgery, radiotherapy, and chemotherapy, targeted inhibitor therapy has gained significant attention as an effective treatment approach by focusing on specific molecules [4,5]. However, the molecular mechanism underlying cervical cancer development remains incompletely understood, and there is currently no efficient small-molecule targeted therapy available for its treatment. Therefore, further exploration into the pathogenesis of cervical cancer is necessary to identify effective targets for clinical intervention.

Methylation is a widespread modification that occurs after protein synthesis and is essential for regulating protein function. Protein arginine methyltransferase 5 (PRMT5) is a member of the PRMT family and is responsible for catalyzing the methylation of arginine residues in target proteins, using methyl groups sourced from S-adenosylmethionine (SAM) [6,7,8]. PRMT5 participates in multiple cellular functions, such as the modulation of gene expression, RNA processing, and signal transduction pathways. It primarily catalyzes the methylation of monomethylarginine (MMA) and symmetric dimethylarginine (SDMA) on arginine residues within histones, which are essential proteins involved in the packaging of DNA into chromatin structure. By modifying histones such as H3R2me2s, H3R8me2s, or H4R3me2s, PRMT5 can influence chromatin structure and gene expression levels [9,10,11,12,13]. In addition to histones, PRMT5 can methylate other protein targets, such as RNA-binding proteins and transcription factors, thereby affecting their functions. The methylation of arginine residues by PRMT5 can have diverse effects, including the alteration of protein–protein interactions, modulating protein stability, and regulation of protein localization, which play pivotal roles in tumorigenesis and metastasis [14,15,16,17]. However, additional studies are needed to illuminate the fundamental mechanisms involved.

C-X-C motif chemokine ligand 10 (CXCL10), also referred to as interferon gamma-induced protein 10 (IP-10), is a chemokine involved in immune responses and inflammation. As a member of the CXC chemokine family, it is secreted by various cell types, such as monocytes, macrophages, fibroblasts, endotheliocytes, and keratinocytes. CXCL10 has an essential function in recruiting immune cells to areas of infection or inflammation. It binds with its receptor, C-X-C motif chemokine receptor 3 (CXCR3), which is present on the surface of multiple immune cell types, such as natural killer (NK) cells, T cells, and dendritic cells [18]. This interaction triggers cellular responses that result in the recruitment of these immune cells to the site of inflammation [19]. CXCL10 primarily serves to promote the mobilization and activation of T cells and NK cells, especially those participating in the Th1 immune response. Th1 lymphocytes produce interferon-gamma (IFN-γ), which further amplifies CXCL10 expression through a positive feedback loop mechanism. CXCL10 is associated with various diseases, particularly autoimmune disorders like multiple sclerosis, systemic lupus erythematosus, and rheumatoid arthritis. Additionally, CXCL10 has also been found to be elevated in viral infections, such as hepatitis, human immunodeficiency virus (HIV), and influenza [20,21,22]. Furthermore, it participates in certain cancer types by promoting tumor cell migration and invasion. In summary, CXCL10 serves as a significant chemokine that plays a crucial role in mediating immune responses and inflammation, as well as in attracting immune cells to areas impacted by infection or inflammation. Its dysregulation has been implicated in various diseases, highlighting its potential as a target for therapeutic interventions.

The tumor microenvironment is composed of cellular and non-cellular elements found within a tumor, including various immune cells, extracellular matrix, fibroblasts, blood vessels, and signaling molecules. These components exert a significant influence on tumor growth, invasion, and response to therapy [23]. Immune cells in the tumor microenvironment can influence tumor progression by secreting cytokines or expressing co-inhibitory molecules [24]. Additionally, tumor cells have the ability to influence the recruitment and function of immune cells by secreting chemokines and cytokines, or by altering the expression of membrane molecules [25]. Notably, CD8^+^ T cells play a crucial role in the immune system’s defense against infections and cancer by recognizing and eliminating foreign substances [26]. The interaction among T cells, the tumor microenvironment, and PRMT5 represents a complex research area that holds promise for therapeutic interventions aimed at enhancing anti-tumor immune responses. PRMT5 is vital for the development and peripheral homeostasis of CD4^+^ and CD8^+^ T cells, as well as their differentiation into Th17 cell phenotypes within the thymus [27]. Deletion of PRMT5 specifically in T cells leads to a substantial reduction in iNKT cells in the thymus, along with a marked decrease in peripheral CD4^+^ and CD8^+^ T cell populations [28]. Targeted deletion of PRMT5 in T cells mitigates experimental autoimmune encephalomyelitis (EAE) by reducing the influx of inflammatory CD4^+^ T cells into the central nervous system (CNS) [29]. Despite its significance, the role of PRMT5 in T cell migration within the tumor milieu remains underexplored, particularly concerning strategies to modulate PRMT5 activity for enhancing the immune response against tumors. A deeper understanding of these interactions may provide valuable insights for enhancing therapeutic efficacy in cancer management.

Our previous research found that knockdown of PRMT5 could increase the number and function of CD8^+^ T cells, and it has been confirmed that the absence of PRMT5 reprograms the T cell-mediated response by regulating the expression of PD-L1, promoting the anti-tumor immune response in cervical cancer [8]. This study aimed to investigate the role of PRMT5 in shaping the tumor immune microenvironment of cervical cancer, with a specific focus on its regulation of CD8^+^ T cell infiltration. We specifically investigated the role of the PRMT5/CXCL10 axis in this process, which was a mechanism that we had not explored in our previous studies. Analysis of TCGA data revealed a correlation between elevated PRMT5 expression and unfavorable outcomes in patients with cervical cancer. Furthermore, we identified that PRMT5 represses the expression of CXCL10 in cervical cancer cells. Through a series of in vivo experiments utilizing wild-type, CD8 KO, and CXCR3 KO mouse models, we demonstrated that the antitumor effect of PRMT5 knockdown is dependent on CD8^+^ T cells and is mediated by the CXCL10/CXCR3 axis. Finally, we showed that a specific PRMT5 inhibitor, EPZ015666, effectively suppresses tumor growth in vivo. Our findings elucidated a novel mechanism by which PRMT5 promotes immune evasion in cervical cancer and highlight the therapeutic potential of its inhibition. Therefore, we provide a crucial missing link in understanding how PRMT5 inhibition leads from the initial T cell homing to its subsequent antitumor activity, thereby forming an immune “hot” tumor microenvironment. Currently, inhibitors targeting PRMT5 are actively being investigated as potential anti-cancer drugs.

## 2. Materials and Methods

### 2.1. Cell Culture

The mouse cervical cancer cell line U14 cells and the human cervical cancer cell line Siha cells were utilized in this study. The U14 cell line was procured from the National Infrastructure of Cell Line Resource in Beijing. The Siha cell line was stored for this laboratory. U14 cells were cultured in DMEM (HyClone, Logan, UT, USA), while Siha cells were grown in RPMI 1640 medium (Gibco, Grand Island, NY, USA). Both media were supplemented with 10% fetal bovine serum (FBS, HyClone, Logan, UT, USA) and 1% penicillin-streptomycin (Gibco, Grand Island, NY, USA). The cells were incubated at 37 °C in a humidified chamber with 5% CO_2_.

### 2.2. Knockdown Cell Lines

For cell transfection, 293T cells were seeded and subsequently transfected with the control plasmid and PRMT5 knockdown plasmid using Lipofectamine 2000 (Invitrogen, Carlsbad, CA, USA). Following their production and collection, U14 cells or Siha cells were infected with the viruses as needed.

To facilitate screening, cells with PRMT5 knockdown and control cells were isolated using 2 µg/mL puromycin (Sigma-Aldrich, St. Louis, MO, USA). The lentiviral shRNAs targeting mouse PRMT5 and shRNAs targeting human PRMT5 were designed as listed in Table S2.

### 2.3. CFSE Assay

After transfection with shPRMT5, U14 or Siha cells were washed with PBS and then adjusted to a concentration of 1 × 10^6^ cells/mL. Following a 10 min incubation at 37 °C with 5 μM CFSE (Invitrogen, Carlsbad, CA, USA), the cells were subsequently plated in a cell culture dish and allowed to incubate for 72 h. Subsequently, samples were collected for analysis using flow cytometry.

### 2.4. Cell Scratched Assay

Three horizontal lines were marked on the back of the 6-well plate, with each line spaced about 1 cm apart. U14 or Siha cells, with PRMT5 shRNA treatment or untreated, were seeded into the 6-well plate filled with complete culture medium and incubated at 37 °C with 5% CO_2_ until they reached 70–90% confluence. Then, the cell culture medium was discarded, and the straight scratches were marked across each well perpendicular to the horizontal line with the 200 µL pipette tip. Subsequently, the cells were washed three times with PBS to eliminate suspended cells, after which cell medium supplemented with 1% FBS was added to each well. The cells were then cultured and observed at 0 h, 24 h, and 48 h, respectively. Cell migration was assessed by measuring the distance of the unoccupied area under microscope at various time points compared with the original scratch.

### 2.5. Transwell Migration and Invasion Assay

The 8 µm chamber (Corning, Corning, NY, USA) was coated without or with 200 μL Matrigel (Corning, Corning, NY, USA) and was then placed in a 5% CO_2_ incubator at 37 °C for 2 h. U14 or Siha cells, either treated with PRMT5 shRNA or not, were rinsed using PBS and then resuspended in medium without serum at 1 × 10^6^ cells/mL. 250 μL cell suspension was placed on the upper side of the insert, while 750 μL growth media was added to the lower chamber, followed by incubation for 24 h or 36 h. Cells that migrated to the opposite side of the insert were rinsed with PBS, fixed in 4% formaldehyde for 20 min, and finally subjected to staining using 0.1% crystal violet for 20 min. The cells that were attached to the upper surface of the insert were subsequently removed with cotton swabs. Images were captured using a light microscope and analyzed across three randomly selected fields.

### 2.6. Cell Apoptosis Assay

U14 or Siha cells with PRMT5 knockdown, along with control cells, were rinsed with PBS. Cell staining was conducted using 5 µL of Annexin V and 1 µL of 1× propidium iodide (PI) (Invitrogen, Carlsbad, CA, USA), followed by incubation for 15 min at room temperature in the dark. Samples were assessed using flow cytometry using a BD LSRFortessa X20 within 1 h. Data were subsequently analyzed with FlowJo v10 software.

### 2.7. Animal Experiments

Female C57BL/6 mice were sourced from the Comparative Medical Center at Yangzhou University. B6.129P2-Cd8atm1Mak/J (CD8 KO) mice and B6.129P2-Cxcr3^tm1Dgen^/J (CXCR3 KO) mice were acquired from Jackson Laboratory. All mouse-related procedures were carried out in compliance with protocols approved by the Institutional Animal Care and Use Committee of Yangzhou University. Six-week-old mice were chosen for the experiments. Control cells and PRMT5 knockdown U14 cells, each consisting of 100 μL (3 × 10^6^ cells), were injected subcutaneously into the right flank of mice (*n* = 4–5 per group). For the subsequent in vivo therapy assay, an additional 100 μL of U14 cells, amounting to 5 × 10^6^ cells, was injected at the same location. 3 days after the U14 cell inoculation, the intraperitoneal administration of the PRMT5 inhibitor EPZ015666 began on a daily basis. PRMT5 inhibitor EPZ015666 (also known as GSK3235025; Selleckchem, Houston, TX, USA) was utilized in the experiments. Solution was diluted in DMSO and prepared at the specified final concentrations (150 mg/kg and 200 mg/kg) for treatment. Daily measurements of tumor dimensions, specifically length (L) and width (W), were taken using a digital caliper. The tumor volume was determined using the formula V = LW^2^/2, and a threshold of 800 mm^3^ was set to assess mortality in the mice. The survival rate of tumor-bearing mice was calculated, and a survival curve was generated.

### 2.8. RNA Sequencing (RNA-Seq)

Control cells and U14 cells with PRMT5 knockdown were rinsed with ice-cold PBS, followed by the addition of 1 mL Trizol to extract RNA. RNA extraction and RNA sequencing were performed by Shanghai Meiji Biomedical Technology (Shanghai, China) for bioinformatic analysis.

### 2.9. Quantitative Real-Time PCR (Q-PCR)

Total RNA was extracted using Trizol (Invitrogen, Santa Clara, CA, USA). Then, cDNA synthesis from the isolated RNA was performed utilizing the PrimerScript™ RT Reagent kit (Takara, Otsu, Shiga, Japan). The ABI Step One Q-PCR Detection System (Life Technologies, Carlsbad, CA, USA) was used to quantify the expression levels of target genes with the SYBR Green Mix Kit. Normalization of the data was performed using the expression levels reference genes, specifically β-actin or GAPDH. Detailed information on the specific primers employed is provided in Table S2.

### 2.10. Immunoblot Analysis

Tumor cell lines and fresh tumor tissues were harvested. Tissue or cells were lysed using RIPA Lysis Buffer (Beyotime, Shanghai, China) supplemented with 1% PMSF and a protease/phosphatase inhibitor cocktail (Thermo Fisher Scientific, Waltham, MA, USA). The lysates were incubated on ice for 30 min and centrifuged to collect the supernatant. The extraction of cytoplasmic and nuclear protein fractions was performed utilizing NE-PER reagents (78833, Thermo Fisher Scientific, Waltham, MA, USA). Tissue or cells were treated with SDS loading buffer (Beyotime, Shanghai, China) in preparation for electrophoresis. Following this, proteins were transferred to nitrocellulose membranes (Millipore, Billerica, MA, USA). To block nonspecific binding, the membranes were incubated with 5% skim milk. Subsequently, primary antibodies were incubated overnight at 4 °C. The membranes were then exposed to secondary antibodies, followed by the use of an ECL detection kit (Thermo Fisher Scientific, Waltham, MA, USA) for protein bands detection. The antibodies utilized are listed in Table S3.

### 2.11. Flow Cytometry

Fresh tumor tissues were harvested from the transplanted mouse models and mechanically dissociated into homogenate. Then, the homogenate was passed through 100 µm cell strainer to obtain single-cell suspensions. Initially, the cells were incubated with monoclonal antibodies specific to surface markers in a FACS buffer consisting of PBS supplemented with 2% FBS and 1 mM EDTA. To facilitate intracellular cytokine staining, a cell stimulation cocktail (Thermo Fisher Scientific, Waltham, MA, USA) was applied to the cells for 5 h prior to the surface staining. Cell fixation and permeabilization were carried out using either BD Cytofix/Cytoperm buffer (BD Biosciences, Franklin Lakes, NJ, USA) or Foxp3 Transcription Factor Staining Buffer Kits (eBioscience, San Diego, CA, USA). Subsequently, antibodies specific to intracellular cytokines or Foxp3 were utilized for staining the cells. Data acquisition was performed using a BD LSRFortessa X20, and analysis was conducted with FlowJo v10 software. Antibodies details are provided in Table S4.

### 2.12. ELISA

The level of CXCL10 in the supernatant was quantified with an ELISA kit (900-K153, PeproTech, Cranbury, NJ, USA). Pre-coated wells were added blocking buffer for 1 h. Briefly, 100 µL of diluted serum samples (1:10) and standards were dispensed into each well and incubated for 2 h. 100 µL of Horseradish peroxidase (HRP) was applied, followed by a 30 min incubation. After washing the wells, 100 µL ABTS was applied, and the plates were incubated for 30 min. The absorbance readings were taken at 405 nm.

### 2.13. Statistical Analysis

Data are expressed as mean ± SEM, and statistical analyses were conducted with GraphPad Prism 9. A two-tailed unpaired Student’s *t*-test was used to evaluate significance, with a *p*-value below 0.05 considered statistically significant.

## 3. Results

### 3.1. PRMT5 Was Linked to Adverse Prognosis in Patients Diagnosed with Cervical Cancer

**Figure 1:**
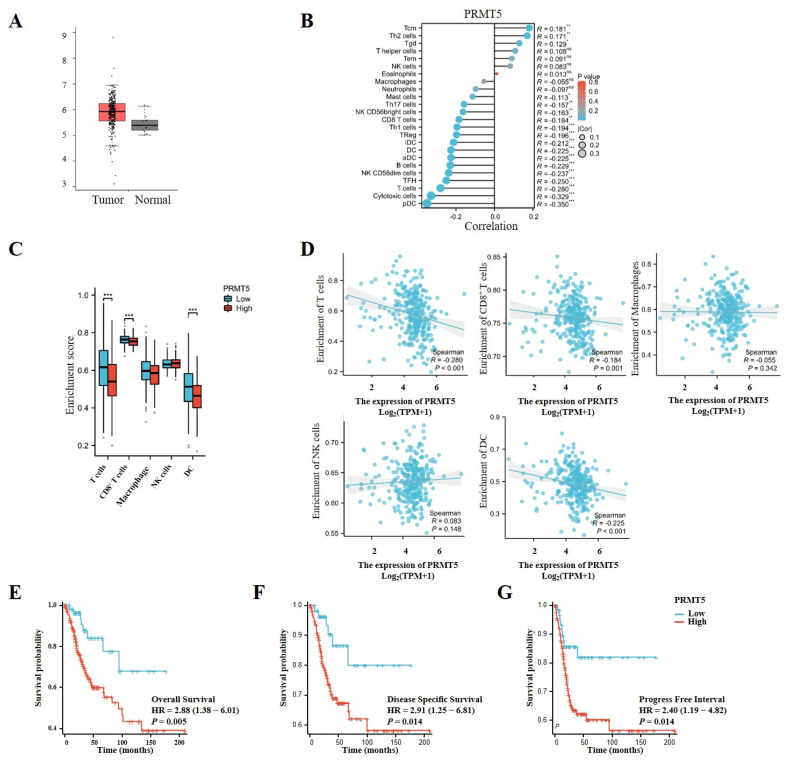
Increases in PRMT5 expression were found to be linked to adverse prognosis in cervical cancer. (**A**) PRMT5 expression was conducted in both healthy cervical tissues and tumor tissues obtained from the TCGA database (num (T) = 306; num (N) = 13). (**B**) Correlation between PRMT5 expression and immune cells from TCGA database (ssgsea test). (**C**) The analyses were performed to investigate the association among high/low PRMT5 expression levels and T cells/CD8^+^ T cells/Macrophages/NK/DC in cervical cancer patients derived from TCGA database. (**D**) The relationship between PRMT5 expression and T cells, CD8^+^ T cells, Macrophages, NK, DC using the TCGA database (ssgsea test). OS (**E**), DSS (**F**) and PFI (**G**) analyses were conducted on cervical cancer patients with high/low levels of PRMT5 using data from the TCGA database. The data represent the mean ± SEM. Blue represents low PRMT5 expression, red represents high PRMT5 expression. ns = no significance, * *p* < 0.05, ** *p* < 0.01, and *** *p* < 0.001.

To explore the potential oncogenic function of PRMT5, we initially evaluated its expression levels in cervical tissues obtained from the TCGA database. PRMT5 expression was observed to be higher in cervical cancer tissues compared to normal tissues, suggesting its role in oncogenesis and its potential as a target for therapeutic target for cervical cancer intervention (Figure 1A). Furthermore, an analysis of TCGA data revealed an inverse relationship between the levels of PRMT5 expression and the infiltration of multiple immune cells, particularly CD8^+^ T cells, as well as plasmacytoid dendritic cells (pDCs) and others (Figure 1B–D). In patients with cervical cancer from the TCGA cohort, higher levels of PRMT5 expression were associated with worse outcomes in overall survival (OS), disease-specific survival (DSS), and progression-free interval (PFI) (Figure 1E–G). Overall, these findings indicate that high levels of PRMT5 expression in cervical cancer are associated with an unfavorable prognosis.

### 3.2. Reduction in PRMT5 Expression Decreased Tumor Migration and Increased the Apoptosis of Cervical Cancer Cells

**Figure 2:**
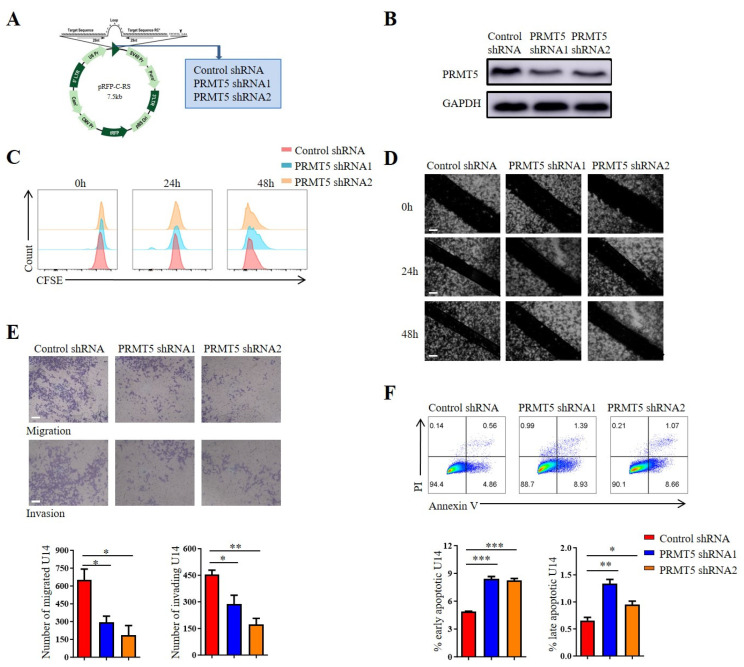
Downregulation of PRMT5 resulted in the inhibition of tumor cell migration, invasion and apoptosis. (**A**) Plasmid vector containing Control shRNA and shRNA targeting PRMT5. (**B**) Comparison of PRMT5 expression between control cells and U14 cells with PRMT5 knockdown. (**C**) Assessment of cell proliferation in U14 cells following PRMT5 knockdown. (**D**) Evaluation of cell migration in U14 cells following PRMT5 knockdown through the Scratch assay. (**E**) Analysis of cell migration and invasion in U14 cells following PRMT5 knockdown through the transwell assay. (**F**) Examination of apoptosis in U14 cells following PRMT5 knockdown. The data represent the mean ± SEM. * *p* < 0.05, ** *p* < 0.01, and *** *p* < 0.001.

Cervical cancer development is strongly associated with the dysregulated proliferation, migration, invasion and apoptosis of cervical epithelial cells. Stable PRMT5 knockdown cell lines were created using U14 cells and Siha cells to explore the role of PRMT5 in the biological characteristics of cervical cancer. It was observed that PRMT5 expression was reduced in silenced cells (Figure 2A,B and Figure S1B). Then, the impact of PRMT5 gene on the migration, proliferation, invasion and apoptosis of cervical cancer cells was investigated, which highlighted its involvement in the initiation and progression of the disease. However, our previous study has shown that PRMT5 did not affect tumor growth [8]. To assess the role of PRMT5 knockdown on tumor cell proliferation, CFSE staining was utilized. Results indicated that the knockdown of PRMT5 did not affect the growth of U14 cells (Figure 2C) and Siha cells (Figure S1C) in vitro, which is consistent with our previous research. Subsequently, we investigate how PRMT5 affects the migratory and invasive capabilities of cervical cancer cells using scratch and transwell assays. Results indicated that PRMT5 reduced tumor cell migration and increased the apoptosis of both U14 cells (Figure 2D–F) and Siha cells (Figure S1D–F) in vitro. The observed impairment in migration and invasion suggested that PRMT5 played a key role in promoting the metastatic potential of cervical cancer cells. Furthermore, the increase in apoptosis upon PRMT5 depletion indicated that it also contributes to tumor cell survival. Taken together, these findings revealed that PRMT5 expression was associated with the migration, invasion and apoptosis of cervical cancer cells, while having no significant impact on their proliferation. This prompted us to hypothesize that the potent antitumor effect of PRMT5 knockdown observed in vivo might not be solely attributable to these direct, cell-intrinsic effects on tumor cells, but likely involves a critical additional component, such as modulation of the tumor immune microenvironment. Nevertheless, these findings were based solely on in vitro experiments, and additional studies are necessary to elucidate how elevated levels of PRMT5 in tumor cells affect the onset and progression of cervical cancer in vivo.

### 3.3. Disruption of PRMT5 Resulted in a Lesser Suppression of Tumor Growth in CD8 KO Mice

**Figure 3:**
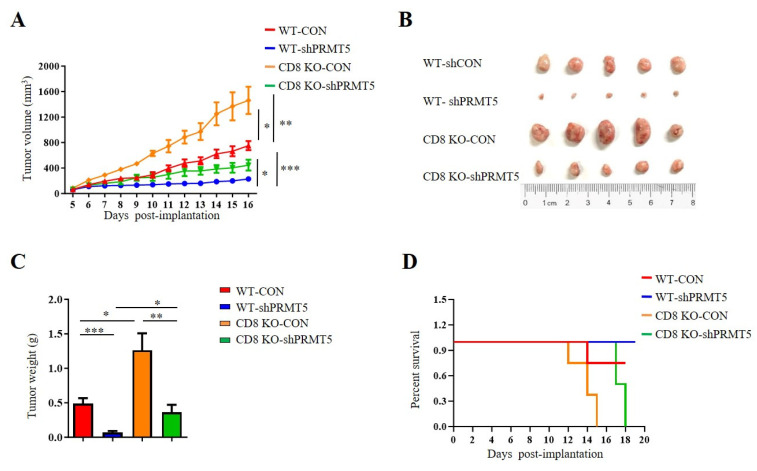
Disruption of PRMT5 suppressed less tumor growth in CD8 KO mice. 6-week-old female C57BL/6 mice and CD8 KO mice received subcutaneous injections of either control cells or U14 cells with PRMT5 knockdown (*n* = 5 per group). (**A**–**C**) The mice were euthanized, and the tumors that were resected were subsequently analyzed on day 16 after inoculation. (**A**) The tumor growth curve observed on the mice is depicted in a line graph. On day 16, images (**B**) and weight measurements (**C**) of the excised tumor were recorded. (**D**) A survival curve was generated for tumor-bearing mice that received subcutaneous injections of control and U14 cells with PRMT5 knockdown. The data represent the mean ± SEM. * *p* < 0.05, ** *p* < 0.01, and *** *p* < 0.001.

T cells, especially CD8^+^ T cells, are essential for antitumor immunity. Therefore, we conducted further investigations to assess whether PRMT5 influenced the effect of CD8^+^ T cells on in vivo tumor growth. To evaluate the role of PRMT5 in cervical cancer development, we constructed tumor models utilizing C57BL/6 mice and CD8 KO mice, which included both control cells and U14 cells with PRMT5 knockdown. Data demonstrated that tumors in the PRMT5 knockdown groups exhibited a significant reduction in both volumes (Figure 3A,B) and weights (Figure 3C), along with a marked survival advantage relative to the control group (Figure 3D). These findings indicated that PRMT5 suppression effectively inhibited the tumorigenic potential of cervical cancer in vivo. Notably, compared to the C57BL/6 mice group, there was a significant increase in tumor volumes and weights in the PRMT5 knockdown groups with CD8 deficiency, which corresponded to lower survival rates than those observed in the control group (Figure 3A–D). These observations indicated that reducing PRMT5 expression in tumor cells may contribute to tumor suppression; however, this effect seems to be diminished in CD8 KO mice.

### 3.4. Disruption of PRMT5 Increased CXCL10 Secretion by Tumor Cells

**Figure 4:**
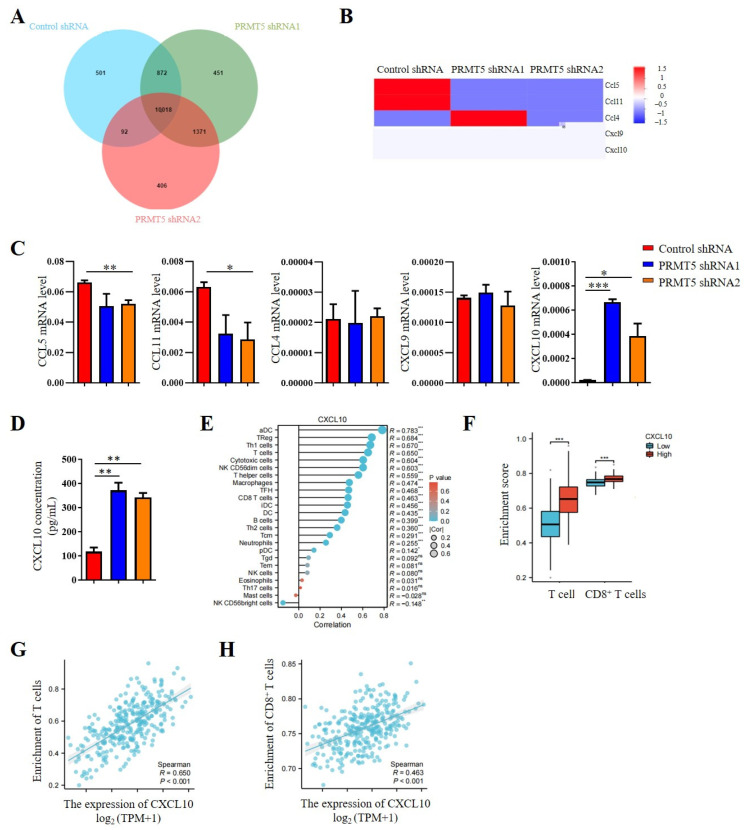
Disruption of PRMT5 enhanced CXCL10 secretion by tumor cells. (**A**) Venn analysis comparing control cells and U14 cells with PRMT5 knockdown using RNA-seq. (**B**) Comparison of chemokine expression profiles between control cells and U14 cells with PRMT5 knockdown analyzed through RNA-seq. (**C**) Real-time PCR experiments were conducted to examine CCL5, CCL11, CCL4, CXCL9, and CXCL10 expression in control cells and U14 cells with PRMT5 knockdown. (**D**) CXCL10 level was measured by ELISA. (**E**) Correlation between CXCL10 expression and immune cell populations was assessed using the Kruskal–Wallis test from TCGA database. (**F**) Analysis of high PRMT5 expression enrichment compared to low PRMT5 expression in T cells and CD8^+^ T cells of cervical cancer patients from TCGA database. Blue represents low CXCL10 expression, red represents high CXCL10 expression. Correlation between CXCL10 expression and T cells (**G**)/CD8^+^ T cells (**H**) was evaluated using the Kruskal–Wallis test from TCGA database. The data represent the mean ± SEM. ns = no significance, * *p* < 0.05, ** *p* < 0.01, and *** *p* < 0.001.

Our previous studies indicated that PRMT5 knockdown increases the CD8^+^ T cells into the tumor tissue. Consequently, chemokine levels in the tumor cells were investigated. The results revealed that PRMT5 deficiency led to elevated expression levels of CXCL10 (Figure 4A,B), which plays a crucial role in attracting CD8^+^ T cells to the tumor site. Moreover, we assessed mRNA expression levels of chemokines in PRMT5 knockdown U14 cells. In light of these findings, our objective was to conduct a more comprehensive investigation into the regulatory function of PRMT5 in the expression of CXCL10. Therefore, we first examined chemokine expression in U14 cells following the knockdown of PRMT5. We observed a decrease in mRNA levels of CCL5 and CCL11 expression, while CCL4 and CXCL9 expression remained unchanged. Notably, CXCL10 expression was indeed significantly increased in U14 cells with PRMT5 knockdown (Figure 4C). Additionally, we observed higher levels of CXCL10 protein in the culture supernatant of U14 cells with PRMT5 knockdown in comparison to the control group (Figure 4D).

Considering the crucial involvement of the CXCL10 pathway in tumor progression, an analysis of data from the TCGA cohort revealed a positive relationship between CXCL10 expression levels and immune cell infiltration (Figure 4E). Cervical cancer patients with high PRMT5 expression can recruit more T cells, especially of CD8^+^ T cells (Figure 4F). In addition, a positive correlation was observed between CXCL10 expression and the inferred abundance of tumor-infiltrating lymphocytes (TILs), suggesting that higher CXCL10 levels are linked to a more immunologically active tumor microenvironment (Figure 4G). Furthermore, we specifically examined the relationship between CXCL10 and cytotoxic T cells. The result revealed a significantly positive correlation between CXCL10 levels and the enrichment of CD8^+^ T cells (Figure 4H). Collectively, these findings identify CXCL10 as a key chemokine upregulated upon PRMT5 deficiency, suggesting that PRMT5 may influence the recruitment of CD8^+^ T cells via the CXCL10 signaling pathway in cervical cancer.

### 3.5. PRMT5 Regulated CD8^+^ T Cells Recruitment Through CXCL10/CXCR3 Axis

**Figure 5:**
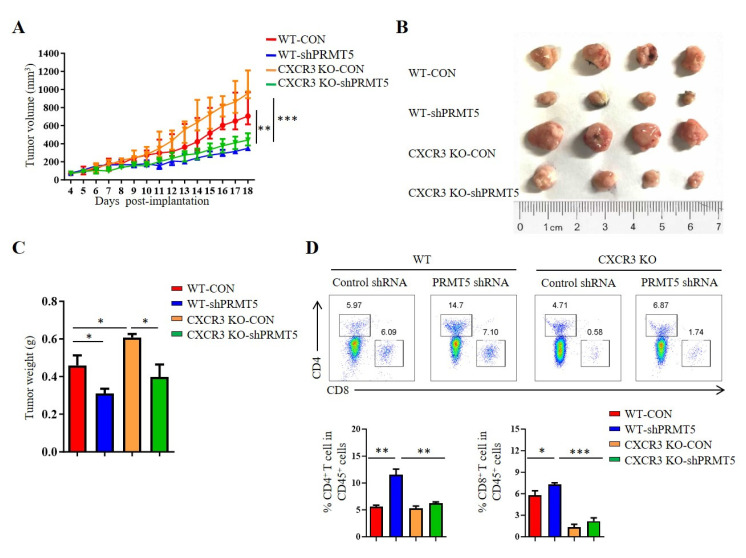
PRMT5 regulated CD8^+^ T cells recruitment through CXCL10/CXCR3 axis. Control cells and PRMT5 knockdown U14 cells were subcutaneously injected into 6-week-old female C57BL/6 mice and CXCR3 KO mice. Mice were euthanized at day 18 after inoculation (*n* = 4 per group). The tumor single cell suspension was prepared and analyzed by flow cytometry. (**A**) A line graph shows the tumor growth curve of mice. Images (**B**) and weight (**C**) of the resected tumor at day 18 after inoculation. (**D**) The percentage of CD4^+^ and CD8^+^ T cells in CD45^+^ cells. The data represent the mean ± SEM. * *p* < 0.05, ** *p* < 0.01, and *** *p* < 0.001.

CXCR3 is a receptor for CXCL10. The CXCL10/CXCR3 signaling pathway is closely related to tumorigenesis [18]. To clarify whether PRMT5 directly regulates the CXCL10/CXCR3 signaling pathway, we established tumor models in CXCR3 KO mice with control cells and PRMT5 knockdown U14 cells. The results showed that the volumes and weights of the tumors in the PRMT5 knockdown groups were significantly increased in CXCR3 KO mice when compared with those of the control group (Figure 5A–C). In C57BL/6 mice, the percentage of CD4^+^ T and CD8^+^ T cells in the tumor microenvironment was significantly elevated in the PRMT5 knockdown group compared to the control group. Conversely, no significant difference in T cell infiltration was observed in CXCR3 KO mice upon PRMT5 knockdown (Figure 5D). Collectively, these findings indicated that the antitumor effect of PRMT5 knockdown is dependent on the CXCL10/CXCR3 axis. Compared to the WT group, the absence of tumor growth inhibition and induced T cell infiltration, alongside the larger tumor size in CXCR3 KO mice, underscores that this chemokine pathway is indispensable for the observed immune-mediated antitumor function.

### 3.6. PRMT5 in the Nucleus Regulated the Recruitment of CD8^+^ T Cells via Symmetric Dimethylation of Histone

**Figure 6:**
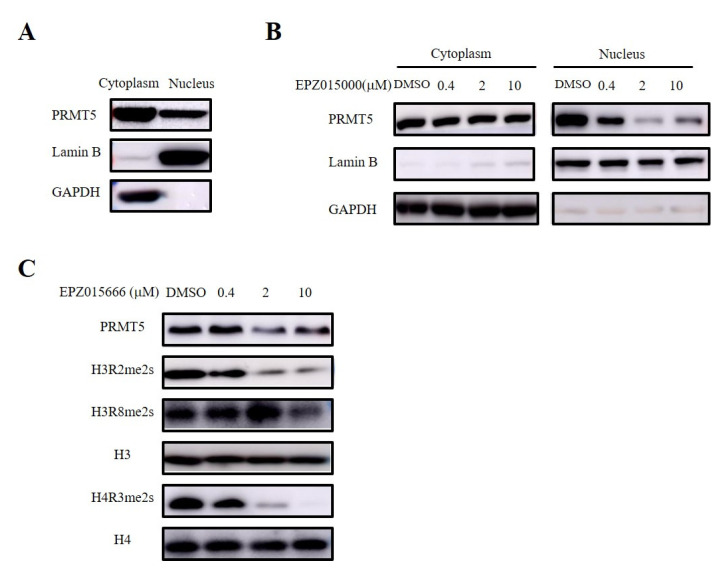
EPZ015666 inhibited dimethylation levels at H3R2, H3R8, and H4R3 sites. (**A**) Analyis of PRMT5 expression in the cytoplasm and nucleus was assessed via Western blot. (**B**) Effect of EPZ015666 on PRMT5 expression in cytoplasm and nucleus by Western blot. (**C**) Impact of EPZ015666 on the expression of PRMT5, H3R2me2s, H3R8me2s, and H4R3me2s in cervical cancer cells was assessed using Western blot. H3 or H4 served as a loading control. Original western blots can be found at Supplementary Materials File 1.

PRMT5 serves as the main type II arginine methyltransferase and is expressed in both the nucleus and the cytoplasm (Figure 6A). Therefore, we validated the site of the antitumor effect of PRMT5 in cervical cancer treatment using a selective inhibitor (EPZ015666) targeting PRMT5. Furthermore, our nucleocytoplasmic separation experiment revealed that EPZ015666 primarily affected the expression of PRMT5 in the nucleus (Figure 6B). PRMT5 is involved in the regulation of gene expression by demethylating histone arginine residues, with symmetric methylation of H3R2, H3R8, and H4R3 being one mechanism involved in this process [8,9,10,11,12]. Our findings indicated that EPZ015666 significantly diminished the symmetric demethylation levels of these histone residues (Figure 6C).

### 3.7. The PRMT5 Inhibitor EPZ015666 Exhibited Significant Antitumor Efficacy Against Cervical Cancer

**Figure 7:**
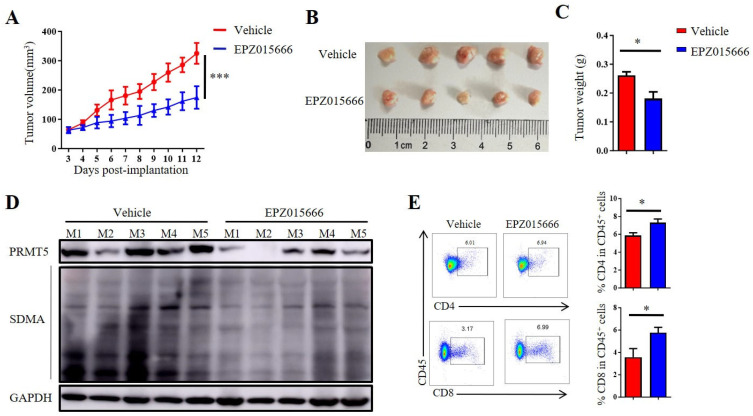

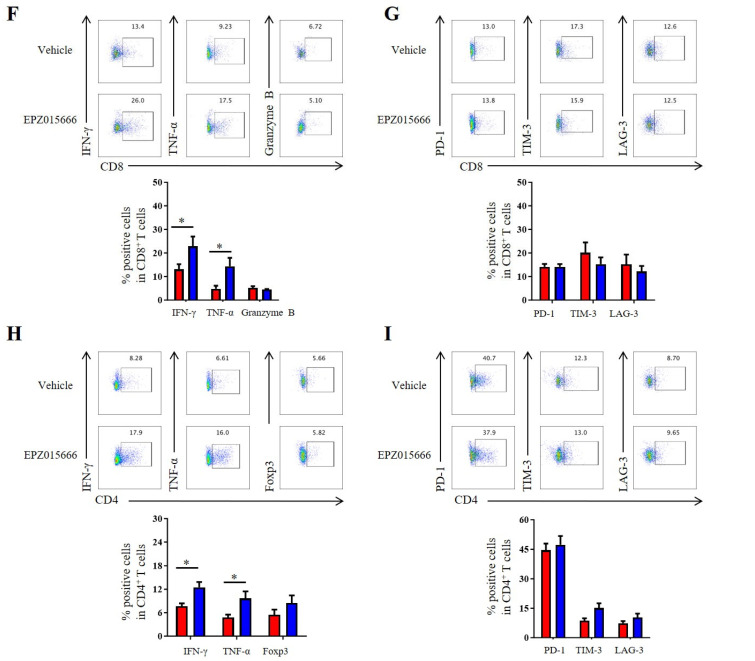
EPZ015666 effectively suppressed cervical cancer growth by enhancing intracellular cytokine expression in T cells. (**A**–**I**) 6-week-old female C57BL/6 mice received daily intraperitoneal injections of EPZ015666 (150 mg kg^−1^) 3 days after inoculation of U14 cells (*n* = 5 per group). (**A**) The growth curve of tumors in the mice is illustrated by a line graph. On day 12 post-inoculation, images (**B**) and weight measurements (**C**) of the excised tumors were collected. (**D**) Analysis was conducted to determine PRMT5 expression and SDMA levels within the tumor. (**E**) The proportion of CD4^+^ and CD8^+^ T cells among CD45^+^ cells was determined. (**F**) IFN-γ, TNF-α and granzyme B expression levels were assessed in CD8^+^ T cells. (**G**) PD-1, TIM-3 and LAG-3 expression levels were revealed through surface examination on CD8^+^ T cells. (**H**) IFN-γ, TNF-α and Foxp3 expression levels were examined in CD4^+^ T cells. (**I**) PD-1, TIM-3 and LAG-3 expression levels was assessed on CD4^+^ T cells. The data represent the mean ± SEM. * *p* < 0.05 and *** *p* < 0.001.

In order to further validate the antitumor potential of PRMT5 deficiency in cervical cancer treatment, we selected the PRMT5 inhibitor EPZ015666 to evaluate its possible therapeutic effects (Figure S2A). The treatment with EPZ015666 resulted in a significant reduction in tumor volume, size and weight in the cervical cancer mouse model (Figure 7A–C and Figure S2B–D), suggesting that EPZ015666 effectively suppressed tumorigenicity in vivo. Additionally, the levels of PRMT5 expression and SDMA were found to be reduced in the EPZ015666 treatment groups (Figure 7D). Relative to the control group, treatment with EPZ015666 led to a significant rise in both the proportion and count of CD4^+^ T and CD8^+^ T cells within the cancer microenvironment (Figure 7E). Furthermore, flow cytometry analysis demonstrated that treatment with EPZ015666 increased CD8^+^ T cells’ secretion of IFN-γ and TNF-α (Figure 7F), while the levels of PD-1 and TIM-3 remained stable (Figure 7G). Similarly, cytokine secretion pattern including IFN-γ, TNF-α and the transcription factor Foxp3 were also enhanced among CD4^+^ T cells with no change observed for checkpoint expression on these cells following EPZ015666 treatment (Figure 7H,I). The results indicated that PRMT5 exerts an intrinsic effect on tumors, enhancing T cell infiltration into the tumor microenvironment while modifying the cytokine expression profiles specific to cervical cancer. To summarize, our findings suggested that targeting PRMT5 could offer a promising therapeutic strategy for the treatment of cervical cancer.

## 4. Discussion

**Figure 8:**
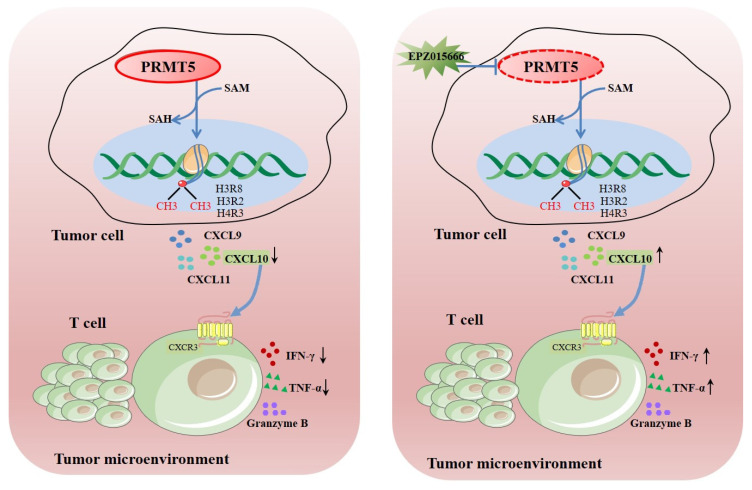
Schematic illustration elucidating the mechanistic function of PRMT5 in facilitating the progression of cervical carcinoma. →: activation effect; ┤: inhibitory effect.

PRMT5 functions as a vital enzyme that is involved in protein arginine methylation, significantly influencing cellular function and human health. Dysregulation of PRMT5 may significantly impact the migration of T cells that are present in the tumor microenvironment. Ongoing research aims to uncover its precise roles and potential therapeutic applications. This study revealed that knockdown of PRMT5 in cervical cancer cells resulted in elevated CXCL10 expression, which was associated with a higher accumulation of CD8^+^ T cells in the surrounding tumor microenvironment (Figure 8). This demonstrated that PRMT5 knockdown induced an antitumor response through regulation of CXCL10 expression and suggested that PRMT5 inhibitors could serve as a promising therapeutic strategy for cervical cancer treatment.

Cervical cancer is primarily associated with human papillomavirus (HPV) infection. Previous studies have reported that PRMT5 promotes replication of infectious bursal disease virus (IBDV) through post-translational modification of HBV [30], but the regulatory mechanisms of PRMT5 in HPV infection have not been fully elucidated. PRMT5 plays a role in multiple biological processes and is associated with several human diseases. It has been linked to a key regulator of cancer development, as its overexpression has been observed in certain types of tumors, and its dysregulation can contribute to abnormal cell change. In colorectal cancer, PRMT5 facilitates the metastasis of tumor cells through the methylation of SMAD4 [31]. Therefore, we investigated the impact of PRMT5 expression on migration and apoptosis in a cervical cancer cell line.

Additionally, there are indications that PRMT5 may alter immune resistance in breast cancer and exhibit an inverse correlation with antitumor immunity in melanoma [32,33]. In our previous study using a mouse cervical cancer model, silencing PRMT5 in tumor cells not only inhibited tumor growth but also elevated both the proportion and count of CD4^+^ and CD8^+^ T cells [8]. The reasons for this increase could be attributed to two possibilities: Firstly, it is possible that PRMT5 regulates T cell proliferation since our previous study demonstrated that no effect on T cell proliferation within the tumor microenvironment upon deficiency of PRMT5 in tumor cells [8]. Secondly, it is plausible that PRMT5 can regulate the secretion of chemokines by tumor cells to recruit peripheral T cells into the tumor microenvironment, which requires further exploration.

Subsequently, we delved deeper into investigating the mechanism underlying antitumor immunity resulting from deficiency of PRMT5. Interestingly, we discovered that PRMT5 deficiency led to a decrease in CXCL10 expression in tumor cells. As reported, CXCL10/CXCR3 axis plays a crucial role as chemotactic molecule [18,19]. Chemokines secreted by tumor cells interact with specific receptors on T cells, facilitating their attraction and recruitment into tumors. In the PRMT5-knockdown alone model, PRMT5 knockdown tumor cells secrete high levels of CXCL10. This chemokine acts as a beacon, recruiting CXCR3-expressing CD8^+^ T cells from the host into the tumor. These recruited T cells then efficiently recognize and kill the tumor cells. The potent immune-mediated cell killing outweighs the direct pro-apoptotic effect, resulting in a net suppression of tumor growth. In the dual-knockdown (PRMT5 and CXCR3) model, while the tumor cells still have PRMT5 knockdown, the host’s T cells lack the CXCR3 receptor. Therefore, they cannot respond to the CXCL10 signal and are not recruited to the tumor site. Without this crucial T-cell infiltration, the immune-mediated killing is abolished. The remaining, weaker pro-apoptotic effect is insufficient to control tumor growth on its own, allowing the tumors to progress and become larger. In summary, the dual-knockdown experiment uncovers that disruption of the CXCL10/CXCR3 signaling pathway can decrease T cell recruitment. The present study revealed that knockdown of PRMT5 leads to an upregulation in the mRNA and protein levels of CXCL10 secretion. The observed increase in CXCL10 mRNA may be due to enhanced transcriptional activation, which could be because the inhibitory effect of PRMT5 on its promoter has been relieved, or due to post-transcriptional mechanisms, such as an increase in mRNA stability. However, the exact underlying mechanism still needs to be further clarified. These findings indicated that PRMT5 deficiency in cervical cancer cells boosts T cell recruitment into the tumor microenvironment by enhancing CXCL10 secretion, resulting in a higher number of T cell infiltrations.

PRMT5 inhibitors are a class of compounds that specifically target the PRMT5 enzyme. Inhibition of PRMT5 can affect various cellular processes and has been extensively studied for its potential in cancer therapy [34]. Research on PRMT5 inhibitors is ongoing in the field of oncology and other areas. In addition, several small molecules have been developed to target PRMT5, including EPZ015666, C220, GSK3326595, JNJ-64619178, PF-06939999 and PR5-LL-CM01, with some showing promise in preclinical studies and clinical trials [35,36,37,38,39]. These inhibitors are under investigation for their ability to target and eliminate cancer cells, particularly those with specific genetic alterations that make them vulnerable to PRMT5 inhibition. In our study, we utilized EPZ015666 (also known as GSK3235025), a highly selective inhibitor of PRMT5, to treat cervical cancer transplanted tumor model, which showed significant antitumor activity. Further investigations are warranted to fully elucidate the therapeutic effect of EPZ015666. Given the crucial role of T cells within the tumor microenvironment, we evaluated cytokine production by CD4^+^ and CD8^+^ T cells that infiltrate the tumor. Our findings indicated that the knockdown of PRMT5 in the tumor microenvironment resulted in increased levels of IFN-γ and TNF-α expression in both CD4^+^ and CD8^+^ T cells. Additionally, we evaluated the expression of coinhibitory molecules PD-1, TIM-3, and LAG-3 on T cells but observed no significant changes in their levels. To summarize our findings concisely: we have uncovered a novel mechanism through which PRMT5 promotes tumor growth, namely by regulating CXCL10 expression within tumor cells while facilitating recruitment of T cells via interaction between CXCL10 and CXCR3. Further investigation is required to assess the effectiveness of PRMT5 inhibitors in patients diagnosed with cervical cancer.

In conclusion, our research results are of great significance for the treatment of cervical cancer. Firstly, they provide a strong basis for targeting PRMT5, which not only directly damages the functions of cancer cells but also enhances antitumor immunity by enhancing the infiltration of CD8^+^ T cells into the tumor microenvironment. This dual mechanism indicates that PRMT5 inhibitors may be effective for “cold” tumors that are currently resistant to immunotherapy. Secondly, the identified PRMT5/CXCL10 axis provides a potential biomarker strategy; patients with high PRMT5 expression and low CXCL10 expression may be ideal candidates for PRMT5 inhibitor treatment. Additionally, it is possible to explore the combination of PRMT5 inhibitors with immune checkpoint blockers (such as anti-PD-1 antibodies) to achieve a synergistic effect. In our model, the potent antitumor activity of the specific inhibitor EPZ015666 highlights the translational potential of this approach and emphasizes the importance of further clinical research. In the current study, we focused on elucidating the novel mechanism of the PRMT5/CXCL10 axis in vivo, primarily utilizing mouse models and lacking a direct relevance to human cervical cancer. Additionally, we indeed not directly test EPZ015666 on primary human immune cells, and we acknowledge that this limitation needs further exploration. In addition, elevated PRMT5 expression in cervical cancer promotes the epithelial–mesenchymal transition (EMT) process, and prior studies have also reported overexpression of other PRMT family members in various cancers [40,41,42]. However, this study did not examine the expression or function of additional PRMT isoforms, highlighting the need for further investigations into potential crosstalk among these PRMT family members. Moreover, it has been reported that several PRMT5 inhibitors are currently under development, and new compounds are continuously being explored for their therapeutic efficacy and safety in targeting PRMT5. Nevertheless, this study provides a theoretical foundation for employing PRMT5 inhibitors as a therapeutic strategy against cervical cancer.

## 5. Conclusions

As revealed by this study, the antitumor effect in cervical cancer cells is linked to the silencing of PRMT5, which promotes T cell recruitment and regulates CXCL10 expression. Notably, a significant correlation was found between elevated PRMT5 expression and poor survival outcomes in cervical cancer patients, underscoring its potential role in promoting tumorigenesis. Furthermore, the PRMT5 inhibitor demonstrated significant efficacy in cervical cancer using a mouse model. These results indicate that targeting PRMT5 may represent a promising strategy in clinical treatment.

## Data Availability

Data are contained within the article or Supplementary Materials.

## Supplementary Materials

The following supporting information can be downloaded at: https://www.mdpi.com/article/10.3390/biom15121717/s1, Figure S1: Downregulation of PRMT5 inhibited tumor cell migration, invasion and apoptosis; Figure S2: EPZ015666 suppressed cervical cancer growth; Table S1: PRMT5 shRNA sequences.; Table S2: Primer sequences for quantitative real-time PCR; Table S3: Antibodies used in the experiment; Table S4: Antibodies used in the experiment. Original western blots can be found at Supplementary Materials File 1.
