# Supplementary material for: Enhancing CD8+ T Cells Infiltration Through the Protein Arginine Methyltransferase 5 (PRMT5)/CXCL10 Axis Restricts Cervical Cancer Progression
Source: Biomolecules. 2025 Dec 10;15(12):1717. doi: 10.3390/biom15121717 (PMC12731132; doi:10.3390/biom15121717)
Supplement: Supplementary file 1 [file biomolecules-15-01717-s001.zip › biomolecules-3984162-supplementary.pdf]

biomolecules

MDPI

Supplementary Materials for

Enhancing CD8⁺ T Cells Infiltration Through the Protein Arginine Methyltransferase 5 (PRMT5)/CXCL10 Axis Restricts Cervical Cancer Progression

Yongshuai Jiang [1,2,3,†,*], Yingying Wei [1,†], Ziyang Li [1], Zhenghang Huang [1], Junsheng Dong [3,4], Weijuan Gong [1,2,3] and Li Qian [1,2,3,*]

[1] School of Basic Medical Sciences & School of Public Health, Faculty of Medicine, Yangzhou University, Yangzhou 225009, China; ysjiang0225@yzu.edu.cn (Y.J.); 18140735925@163.com (Y.W.); yzucris@gmail.com (Z.L.); zhenghanghuang32@gmail.com (Z.H.)
[2] Key Laboratory of the Jiangsu Higher Education Institutions for Nucleic Acid & Cell Fate Regulation, Yangzhou University, Yangzhou 225009, China
[3] Jiangsu Key Laboratory of Zoonosis, Yangzhou University, Yangzhou 225009, China
[4] College of Veterinary Medicine, Yangzhou University, Yangzhou 225009, China
* Correspondence: junsheng@yzu.edu.cn (J.D.); wjgong@yzu.edu.cn (W.G.); qianl@yzu.edu.cn (L.Q.)
† These authors contributed equally to this article.

This file includes:

Figures S1 to S2
Tables S1 to S4

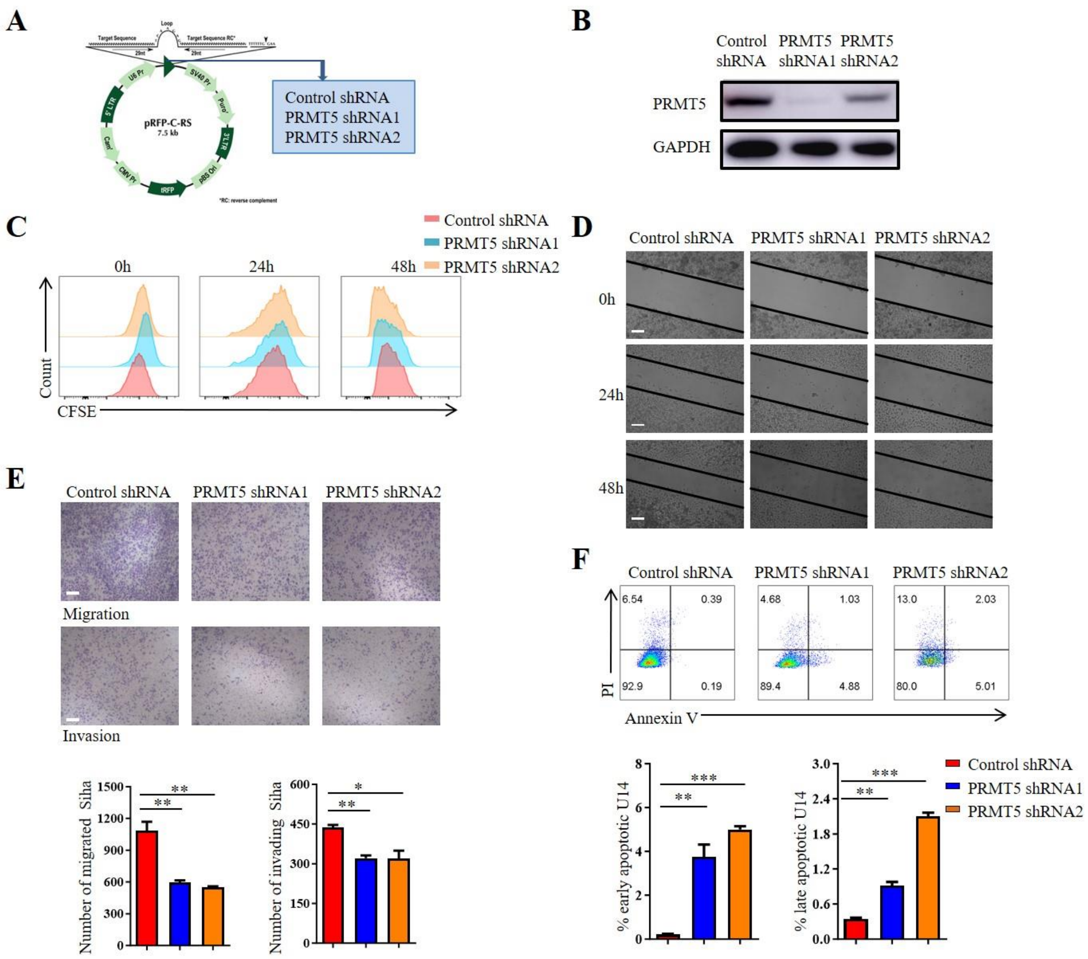

Supplementary Figure 1. Downregulation of PRMT5 inhibited tumor cell migration, invasion and apoptosis. (**A**) Plasmid vector containing Control shRNA and shRNA targeting PRMT5. (**B**) Comparison of PRMT5 expression between control cells and Siha cells with PRMT5 knockdown. (**C**) Assessment of cell proliferation in Siha cells following PRMT5 knockdown. (**D**) Evaluation of cell migration in Siha cells following PRMT5 knockdown through Scratch assay. (**E**) Analysis of cell migration and invasion in Siha cells following PRMT5 knockdown through transwell assay. (**F**) Examination of apoptosis in control cells and Siha cells with PRMT5 knockdown. The data represent the mean ± SEM. *$P < 0.05$, **$P < 0.01$, and ***$P < 0.001$.

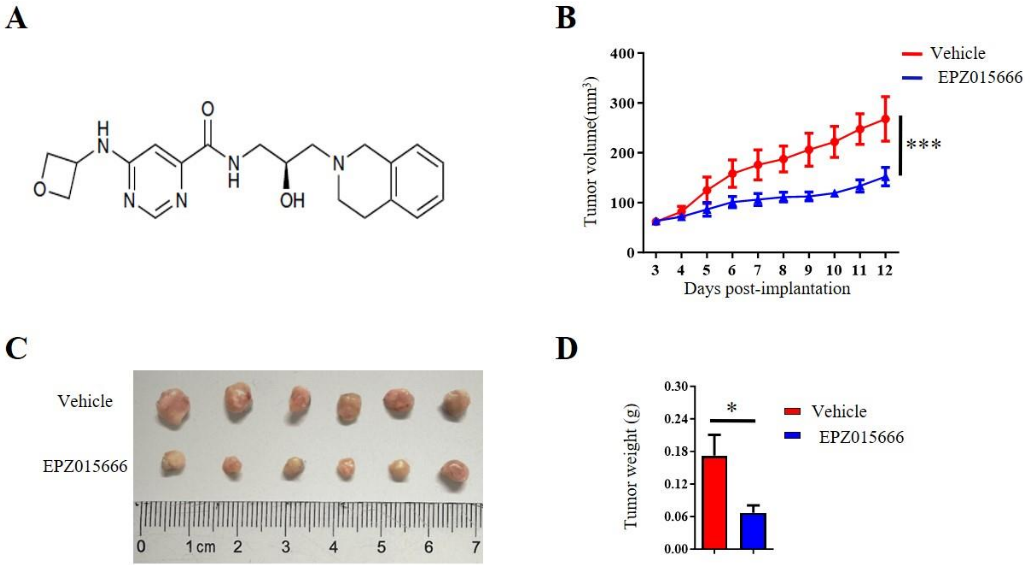

Supplementary Figure 2. EPZ015666 suppressed cervical cancer growth. (**A**) The structure of EPZ015666. (**B-D**) On day 3 after inoculation of U14 cells, 6-week-old female C57BL/6 mice (n = 6 per group) received daily intraperitoneally injections of EPZ015666 (200 mg kg^{-1}). (**B**) The tumor growth curve of the mice is illustrated in a line graph. On day 12 post-inoculation, images **(C)** and weight measurements **(D)** of the excised tumors were recorded. The data represent the mean ± SEM. *P < 0.05 and *** P < 0.001.

Supplementary Table 1. PRMT5 shRNA sequences.

mPRMT5 shRNA1	5’- AGCCAGGTGACAGTTGTCTCATCAGACAT -3’
mPRMT5 shRNA2	5’- TTCCTGTGGAGGTGAACACGGTGCTTCAT -3’
hPRMT5 shRNA1	5’- GGCTCAAGCCACCAATCTATG -3’
hPRMT5 shRNA2	5’- GCCCAGTTTGAGATGCCTTAT -3’

Supplementary Table 2. Primer sequences for quantitative real-time PCR.

hGAPDH	Forward	5’- GGTGGTCTCCTCTGACTTCAACA -3’
	Reverse	5’- GTTGCTGTAGCCAAATTCGTTGT -3’
hPRMT5	Forward	5’- CTGTCTTCCATCCGCGTTTCA -3’
	Reverse	5’- GCAGTAGGTCTGATCGTGTCTG -3’
mβ-actin	Forward	5’- TGTCCACCTTCCAGCAGATGT -3’
	Reverse	5’- AGCTCAGTAACAGTCCGCCTAG -3’
mPRMT5	Forward	5’- CTGAATTGCGTCCCCGAAATA -3’
	Reverse	5’- AGGTTCCTGAATGAACTCCCT -3’
mCXCL10	Forward	5’- CCAAGTGCTGCGTCATTTTC -3’
	Reverse	5’- GGCTCGCAGGGATGATTTCAA -3’
mCXCL9	Forward	5’- GGAGTTCGAGGAACCCTAGTG -3’
	Reverse	5’- GGGATTTGTAGTGGATCGTGC -3’
mCCL4	Forward	5’- TTCCTGCTGTTTCTCTTACACCT -3’
	Reverse	5’- CTGTCTGCCTCTTTTGGTCAG -3’
mCCL5	Forward	5’- TTTGCCTACCTCTCCCTCG -3’
	Reverse	5’- CGACTGCAAGATTGGAGCACT -3’
mCCL11	Forward	5’- GAATCACCAACAACAGATGCAC -3’
	Reverse	5’- ATCCTGGACCCACTTCTTCTT -3’

Supplementary Table 3. Antibodies used in the experiment.

Antibodies	Source	Identifier
Anti-PRMT5 Antibody	Abcam	ab109451
Anti-SDMA Antibody	Cell Signaling Technology	13222S
Anti-GAPDH Monoclonal Antibody	Absin	abs830030
Anti-Lamin B Antibody	Proteintech	66095-1-Ig
Anti-Histone H3 Antibody	Abcam	ab1791
Anti-Histone H3R2me2s Antibody	Abcam	ab194684
Anti- Histone H3R8me2s Antibody	EpiGentek	A-3706
Anti- Histone H4R3me2s Antibody	Abcam	ab5823
Anti-Histone H4 Antibody	Cell Signaling Technology	2935S

Supplementary Table 4. Antibodies used in the experiment.

Antibodies	Source	Identifier
Monoclonal Antibody CD45 PerCP-Cyanine5.5	eBioscience	45-0451-82
Rat Anti-Mouse CD45 BV421	BD	563890
Monoclonal Antibody CD3 Alexa Fluor 700	eBioscience	56-0032-82
Monoclonal Antibody CD4 FITC	eBioscience	11-0041-85
Rat Anti-Mouse CD8a PE-CF594	BD	562283
Hamster Anti-Mouse CD279 (PD-1) BV421	BD	562584

Anti-mouse Antibody CD366 (TIM-3) BV605	Biolegend	119721
Rat Anti-Mouse CD223 (LAG-3) BV711	BD	563179
Monoclonal Antibody IFN-γ PE-Cyanine7	eBioscience	25-7311-82
Monoclonal Antibody TNF-α APC	eBioscience	17-7321-81
Monoclonal Antibody Foxp3 APC	eBioscience	17-5773-82
Monoclonal Antibody granzyme B PE	eBioscience	12-8898-80
Fixable Viability Dye eFluor 780	eBioscience	65-0865-18
LIVE/DEAD™ Fixable Blue Dead Cell Stain Kit	Life	L34962
